# Optimising peak energy reduction in networks of buildings

**DOI:** 10.1038/s41598-024-52676-2

**Published:** 2024-02-16

**Authors:** A. Poghosyan, N. McCullen, S. Natarajan

**Affiliations:** Centre for Regenerative Design & Engineering for a Net Positive World, University of Bath, Claverton Down, Bath, BA2 7AY UK

**Keywords:** Energy grids and networks, Civil engineering

## Abstract

Buildings are amongst the world’s largest energy consumers and simultaneous peaks in demand from networks of buildings can decrease electricity system stability. Current mitigation measures either entail wasteful supply-side over-specification or complex centralised demand-side control. Hence, a simple schema is developed for decentralised, self-organising building-to-building load coordination that requires very little information exchange and no top-down management—analogous to other complex systems with short range interactions, such as coordination between flocks of birds or synchronisation in fireflies. Numerical and experimental results reveal that a high degree of peak flattening can be achieved using surprisingly small load-coordination networks. The optimum reductions achieved by the simple schema can outperform existing techniques, giving substantial peak-reductions as well as being remarkably robust to changes in other system parameters such as the interaction network topology. This not only demonstrates that significant reductions in network peaks are achievable using remarkably simple control systems but also reveals interesting theoretical results and new insights which will be of great interest to the complexity and network science communities.

## Introduction

We spend nine-tenths of our lives within buildings, relying on a consistent supply of energy to live and work well. Buildings not only account for between 40 and 60% of global energy demand^1–6^, but are also responsible for creating sharp peaks in demand in response to extreme heat or cold^7–9^. Such peaks create load on the grid that can result in cascading failure of the generators^10^. Recent examples—such as the blackout in Texas in February 2021 due to extreme cold, which left more than 10 million people without power for days^11^, followed by a June 2021 blackout due to extreme heat—demonstrate the severe stress on energy networks emanating from unprecedented extreme events, which are only expected to increase in frequency and severity as the global climate warms^12–20^. Even predictable extremes are unsustainable in the long term, as they require a reserve of back-up generators (sometimes referred to as “peakers”) which are expensive and opposed to current climate change mitigation aspirations. This is significant as just 1% of annual hours are responsible for between 10 and 20% of wholesale electricity costs in the U.S.^21^, reflective of the need for more expensive and carbon-intensive peakers such as coal or gas fired generators.

Hence, it is of the utmost importance that energy systems are designed to be not only resilient, but cost-effective and carbon efficient, now and in the future. Since supply-side solutions can aid resilience, but are expensive^22^ and result in greater carbon emissions^23,24^, there is a strong focus on demand-side management (DSM) strategies, which tackle the problem of peak demand at the building and user level. These include techno-economic strategies for dispatchable loads—those that can respond to changes in a short timescale of typically less than 30 min^25^—and tariff-driven strategies for non-dispatchable loads^26,27^. Unfortunately, despite considerable recent interest, the maximum peak reduction of most current strategies has been shown to be only around 5%^28^, with only a few indicating larger potential reductions^29,30^. The two main challenges in previous strategies have been insufficient user-engagement to realise savings^28,31^ and the need to predict when loads might occur^25–27,32–34^. The latter often relies on hard-to-obtain data—including appliance inventories, scheduling, the timing and size of actual loads, occupancy and localised weather—further complicating the prediction problem. There is hence an urgent need to discover what the theoretical limit might be to minimal *peakiness* and to what extent this limit might be practically achieved. The current work aims to determine the best values for the control parameters in an idealised energy consumer model that result in optimal peak flattening.

Another significant advancement that could be made to the majority of demand-side approaches is that they currently often only consider how peak loads can be reduced at the level of an individual building. This means that it is possible that several buildings in a network may end up shifting loads to a different time of day simultaneously—leading to new peaks and thus reducing the overall efficacy of the measure^35^. Given that grid-level failures can occur due to the synchronisation of peak loads across several buildings in a network, substantial peak reductions could be achieved by considering how loads, especially those of a similar type, can be coordinated across groups of buildings. Even techniques that approach the problem at group level usually centralise the optimisation and control schemes^25,26,34–39^. This imposes computational complexity and a higher associated cost, which increases exponentially with the number of coordinated buildings^35^, as identifying optima often requires considering the entire search space^40^. This significantly limits the potential scale of application given that the number of buildings served by a single network end-point are usually at least an order of magnitude higher than can be studied effectively^41^.

Hence, an ideal peak reduction strategy would be one that (i) allows decentralised load coordination between buildings such that the coincidence of loads is minimised, (ii) is computationally simple, (iii) is easily scalable to a large number of buildings, (iv) has the potential to be low-cost and (v) requires little to no human intervention. A similar set of requirements is described in^42^, which provides a strong motivation for the current approach.

The natural world has many examples where complex systems are governed by interactions between small numbers of individuals within a much larger system. There are many powerful examples of how decentralised coordination between elements of a complex biological system—with no knowledge of the overall system’s state or properties—can result in highly desirable “emergent” behaviour at system level^43,44^. Notable examples include models of starling *murmurations*, showing that the birds only need to interact with six or seven close neighbours to achieve startling coordination of the whole flock^45,46^, as well as synchronisation of flashing fireflies^47^ which react to local stimuli. Such bio-inspired approaches have been used to solve problems in crowd disaster and pedestrian flows^48^, collective learning^49^ and flight formation control of air vehicles^50^. Inspired by these, the current work focuses on the applicability of a local interaction model to the problem of energy demand management, to determine the best achievable efficacy in achieving global coordination and load management at the system level.

A simple schema is introduced and numerically implemented, which focuses on small local groups (or *networks*) of individual buildings sharing minimal information. This model is used to study the effect of the interaction-network structure and model parameters that could result in effective load-flattening behaviour using this type of, ideally automated, decentralised self-organising load coordination between small groups of buildings. The extent of the peak load reduction that could be achieved through such schemes is also investigated, in order to determine the optimum flattening corresponding to the minimum *peakiness* of the loads (see supplementary Video 1 an illustrative example of a peaky load and Video 2 for a flattened load). The model is similar in form to other work studying coordination amongst distributed energy resources^51^, but the focus here is on loads in individual buildings. Dwellings are used to inform the example building loads in the model due to their larger energy demand profile compared to non-dwellings and the fact they present a more significant coordination challenge due to the distributed nature of loads. The coordination schema is based around loads that are “shiftable” in time^52–54^, as opposed to base loads (e.g., stand-by appliances, routers etc.) and on-demand loads (e.g., kettles and televisions). After initially considering the less-constrained shiftable loads, such as dishwashers, the model is extended to more time-constrained thermal loads, such as those for space heating and hot water. Loads from heating and cooling systems in buildings can be large. For example, a gas boiler or electric heat pump has a rated capacity about five times that of a typical large home appliance such as a dryer; and a domestic air-conditioning unit about twice as large as a typical appliance. Such loads are known to have a significant impact on network peaks^55^, due to both the size of the loads and their constrained timing. This impact on network peaks can also be particularly significant given their interaction with external weather conditions and heat losses in traditional, poorly insulated, buildings. However, in modern and future building stock, the heat-loss time constants on such loads will be extended by improved insulation and air tightness, so these will also become more shiftable in time. While the thermal loads may also reduce in magnitude as a consequence, this is not guaranteed^56^ and the problem of peak demand will likely remain. The main focus of the current work is on demonstrating the intrinsic effectiveness of the peak reduction schema and finding the algorithm and interaction network parameters that achieve the best results.

An agent-based modelling (ABM) framework was used to run a variety of simulations and determine which key parameters significantly influence the magnitude of any observed peak reductions arising from the schema. The factors investigated were group size, interaction network topology, coordination time-scale and the size of load allowed to be redistributed in each time-step. Each of these factors represents a significant unknown that could affect the overall robustness of the system: for example large groups may prove harder to coordinate in practice, a single successful network topology could be less flexible than a multitude of topologies and longer coordination time-scales might negatively affect user-acceptance depending on the nature of the load. In addition to the numerical study, a small physical system was constructed to test the effectiveness of the schema under realistic conditions.

## Methodology

### Overview

In this section, the methods used for the numerical and experimental results are described. First, in Section “A simple schema for load coordination”, the simple peak coordination schema and algorithm used to implement it are introduced. Next, in Section “Network topologies” the different network topologies that were investigated are detailed. This is followed in Section “Modelling approach” by a discussion of the modelling approaches that can be used represent the load sharing schema, with the model set-up and underlying data assumptions given in Setion “Model set-up and implementation”. Finally, the set-up of the physical demonstration is described in Section “Experimental set-up”.

### A simple schema for load coordination

The buildings or dwellings constitute the *nodes* on an interaction network and are directly connected with others (their *network neighbours*) via information links (the *network edges*). Several common network topologies were investigated (described in Section “Network topologies”). For the nodes to coordinate their demand, some information must be exchanged between groups of directly-connected nodes, termed the *neighbourhoods* of the nodes. In this paper, the term ‘node degree’ is used to mean the dwelling and its neighbours, i.e., its *closed neighbourhood*. This information is used to enable one of the following actions—if a suitable shiftable load has been requested for either now or is offset delayed until later:

(i) consume a shiftable load now, to fill spare capacity—either as scheduled or from any previously delayed loads;
(ii) delay the load until later, to reduce current demand;

This forms the minimal set of actions that a node-level agent in the network may take that is expected to be sufficient to flatten load profiles at the system level. While elaborations on these actions are possible—such as “consume $x\%$ less energy now” or “use appliance x but not y”—these can be considered semantic variations on the basic rules. For simplicity the actions are framed in terms of loads, even though energy consumption is in reality never a direct action but rather the result of some other action motivated by the needs and desires for daily living, such as turning on a heating system to increase comfort, watching television, or making tea^57^.

For an agent to take one of the above actions it requires a knowledge of the current neighbourhood load compared to the maximum allowable load at any given time. This can then be used along with its own current and scheduled—i.e., previously unfulfilled—demand, to act to help reduce inter-building peaks. The “network neighbourhood peak” is the peak load, defined as $l_{max}$, expected over a defined period for that neighbourhood. The minimum external information that needs to be transferred to each agent is therefore the load drawn by its neighbourhood at any given point in time. This provides a very simple definition of the information needed by each building for load coordination, involving just two aspects alongside its own demands: the likely neighbourhood peak $l_{max}$ and the current load drawn by a given building’s neighbours. This use of minimal information by individuals mirrors models of flocking birds, where a given individual adjusts its own position and speed based on the relative position of its nearest neighbours^46^.

Once these simple pieces of information are known, the dwelling agent decides both: (a) whether to either delay load consumption to lower current demand or consume scheduled load to fill a gap in demand; and (b) how much load to shift if this is required. Both the threshold for the decision to shift loads and the amount of load to shift are determined as a proportion ($\alpha$) of the permissible peak load ($l_{max}$). The control parameter $\alpha$ acts as a limit to the amount of demand a single actor can shift in one go and prevents multiple buildings inadvertently creating a new peak at the current time-step through coincident rescheduling. The delay is accomplished within a ‘shifting window’, defined as the maximum length of time that loads can be delayed, with the actual delay being randomly selected in the system. Section “Model set-up and implementation” explains how these features are implemented within the peak coordination algorithm.

The ideal network load would be a constant load profile, given by averaging the total network load (for all dwellings) in the network over the time interval being investigated. This is related to $l_{max}$ in that the most extreme scenario would be where all neighbourhoods peak simultaneously, with each using all available loads at the same time. The root mean square error (RMSE) between a given load profile and the ideal average network load is used to compare the un-adjusted load distributions to those using the peak coordination schema. Low values of the RMSE mean that the corresponding load profile is close to the flat average network load—with a totally peak-free, flat profile having an RMSE of zero. In the presented results, the RMSE values are plotted as a function of the parameter $\alpha$, to easily compare them with the other parameters investigated. Ramp-rates—i.e., the maximum rate of change of demand—are presented in half hour intervals, as this is a common network trading period, such as in the UK’s national grid^58^.

### Network topologies

There are a wide range of network topologies described in the literature, of which *partition* and *small-world* topologies (defined in Sections “Random networks and the configuration model” and “Partition networks”) cover all the essential features of real world energy system networks, and hence are commonly used for modelling smart grid communication and control networks^59^. These can be benchmarked against *random* networks that have no inherent clustering into groups. Hence, numerical experiments were run using these three network topologies, across a range of network parameters, in order to compare and assess how they influence the effectiveness of a given control strategy. The networks simulated had a total of 100 nodes (representing 100 dwellings) as a conveniently large number sufficient to contain several groups of neighbours and broadly representative of real networks (the median number of consumers per substation on low voltage electricity distribution systems is approximately 100^41^). However, direct interactions were only between much smaller groups of buildings within this larger system, and hence the results are scalable to any arbitrary size.

The two essential features of any network are its *nodes* and *links* (or edges). In the following description *nodes* represent the dwellings and *links/edges* the connections (or information flows) between them.

#### Random networks and the configuration model

**Figure 1:**
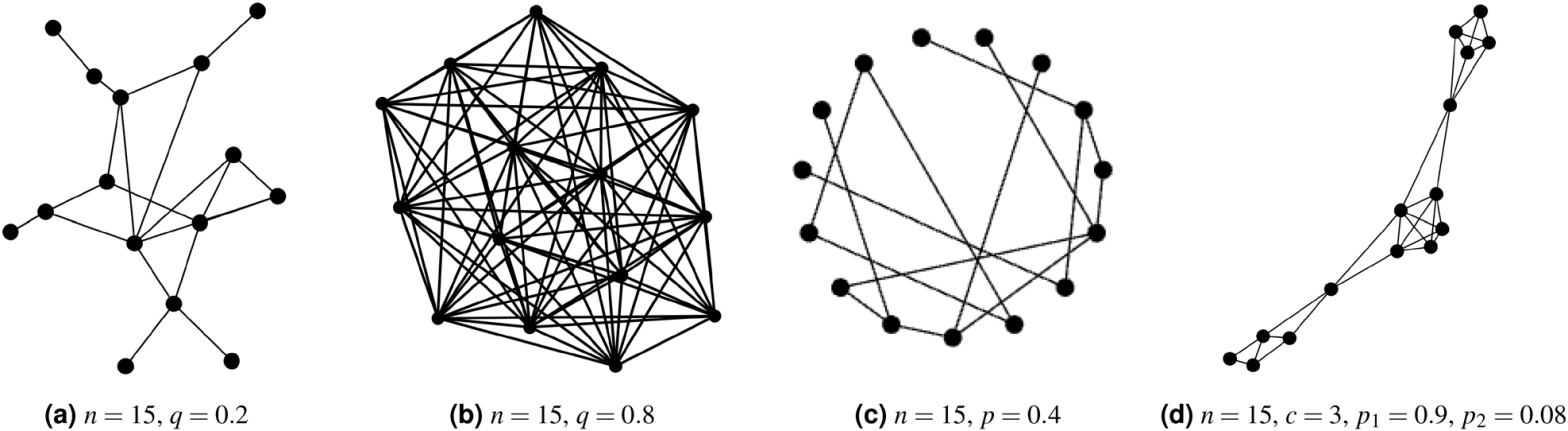
Different network topologies, showing (**a**) & (**b**) examples of random (ER) networks for different choice of link probabilities *q*, (**c**) a Watts–Strogatz *small world* network with local connections and long-range short-cuts and (**d**) a partition network that has intra- and inter-group connections.

Random networks are most commonly generated using Erdős and Renyi’s (ER) random graph model^60^. This is a network with *n* nodes, where each node is linked to another (its *neighbour*) with probability $0 \le q \le 1$. This parameter controls both the density of the network as well as the *degree* of the nodes, defined as the (average) number of links per node. Figure 1a, b illustrate how the value of the probability *q* can affect the structure of random networks.

However, ER networks lack certain important characteristics such as the ability to specify the precise degree for a given node^61^, which is important to carefully control to ensure results are comparable. This aspect of random graph models can be improved by using the configuration model, in which the degrees of nodes are prescribed beforehand^62–64^.

#### Small world networks

Small world networks, generated by the model of Watts and Strogatz (WS), can be used to represent the characteristics of real-world networks with a small number of links connecting any pair of nodes^65^. A small world network of *n* nodes is generated by the following algorithm^65^:

(i) generate a grid with *n* nodes such that the nodes can be arranged in a regular lattice or ring;
(ii) connect each node in the ring to its *d* nearest neighbours (where *d* is an even number for symmetry);
(iii) “rewire” each link in the regular network with probability *p* – i.e., disconnect it from one of its neighbours and connect it with another node that is chosen uniformly at random from the other nodes (often using pairwise swapping to preserve the degree of each node).

Figure 1c illustrates a small world network of $n=15$ nodes, where number of nearest neighbours $d=2$ and probability of rewiring a link is $p=0.4$. When the rewiring probability $p=0$ the network remains a regular lattice with high local clustering^66^ but as rewiring probability increases to $p=1$ the small world network is the same as a random network^64^ with no local structure. Hence WS networks can be used to represent a spectrum of network topologies between these two extremes, with a range of local connectivity.

#### Partition networks

The connectivity can be further controlled to split networks into local groups using the partition network model, which separates nodes into different communities. Two nodes in the same community form a link with probability $0 < p_1 \le 1$ and nodes of different communities are connected with probability $0\le p_2 < 1$, with $p_1 > p_2$ for distinct communities to exist. Figure 1d illustrates a typical partition network with a constant degree $d=4$.

### Modelling approach

There are two alternative design approaches available for modelling complex systems: top-down and bottom-up. In contrast to the top-down approach—which starts with specifying system parameters and outcomes at the macro-scale (often assuming global knowledge of the system) before these are passed down the modelling chain to generate a system response—the bottom-up approach specifies the behaviour of the individual components (nodes), with the global behaviour emerging out of interactions between these components and their environment^67^. Taking inspiration from biological systems, which can coordinate large communities with only local information exchange, the simple schema introduced in Section “A simple schema for load coordination” demonstrates a bottom-up approach, particularly as it involves no centralised control and little knowledge of the wider system by the individual nodes.

Given that the focus here is in the behaviour arising from the interaction of agents (buildings or dwellings) within a system that are capable of taking actions in relation to their local environment (network neighbourhood), an intrinsically bottom-up agent based modelling (ABM) modelling framework was used to implement the scheme. As it is both governed by deterministic rules, as well as using a probabilistic element in the individual runs, it can demonstrate the key outcomes of implementing the schema whilst incorporating the high levels of uncertainty that are present in modelling social phenomena. The main advantage of ABM over other modelling techniques (e.g., purely stochastic modelling or optimisation algorithms) is its ability to allow the study of interactions between components and to discover their emergent collective behaviour^68–70^.

Indeed, since energy systems are considered complex dynamical networks with multiple components that interact, adapt and evolve^71^, several studies have employed ABMs to study energy infrastructure and electricity markets^72–74^, including several DSM strategies. Peak demand reductions envisaged by these DSM studies range between 9 and 17%^27,75–79^ though none, as discussed, consider peak coordination between neighbours.

#### ABM implementation of the peak demand coordinating scheme

An Agent Based Model was employed to investigate the system-level emergent result of scheduling of various shiftable appliance loads in different networks of dwellings for the purpose of optimal peak coordination. The effect of three key aspects on peak reduction were investigated, based on Sections “Network topologies” and “A simple schema for load coordination”:

(i) the effect of network topology, including both the network structure type and the average number of neighbours; (ii) the length of the time window within which a given agent is allowed to shift demand and (iii) the amount of load allowed to be shifted by any single agent, as a proportion $\alpha$ of the peak neighbourhood load $l_{max}$.

Hence, these were carefully controlled within the model.

**Figure 2:**
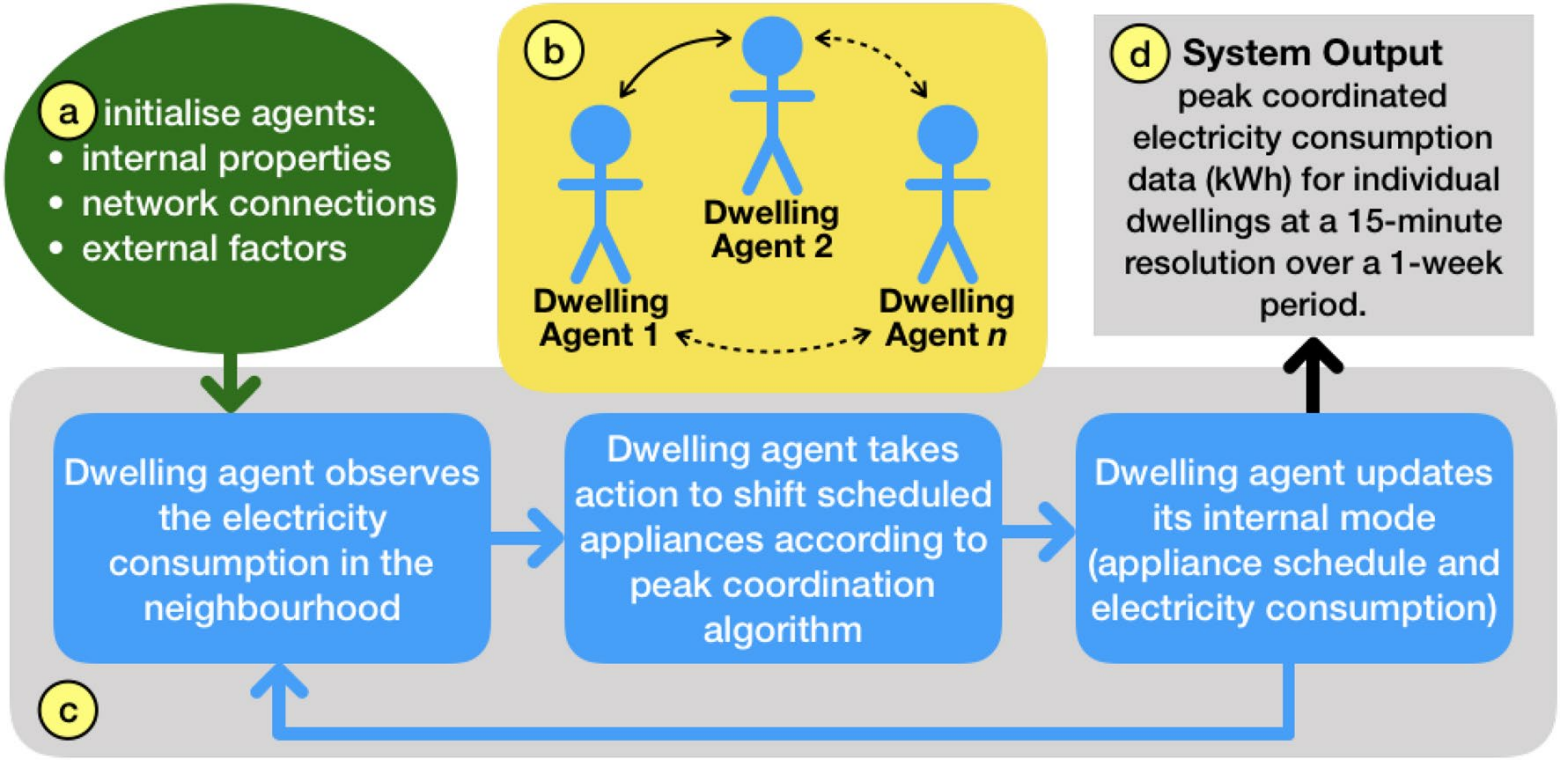
Agent Based Model (ABM) framework, showing Agent initialisation parameters (**a**), set up of the network of links between agents (**b**), the demand shifting routine (**c**) and system-level output (**d**).

Each dwelling in the network is considered an agent with defined properties, as shown in Fig. 2. Once the model is calibrated using input data, with constraints and rules defined in the load coordination schema in Section “A simple schema for load coordination”, the ABM simulation consists of the following steps, as shown in Fig. 2:

- The agents are initialised—each having an initial internal state representing its load profile for the day, as it would be in an uncoordinated state;
- The network environment is set up, choosing the precise network parameters and defining the links for information flows between directly connected neighbours;
- The ABM runs—updating usage every 15 min (an interval often used for real-time physical modelling of electricity networks and analysing peak load behaviour^80–82^)—through a decision cycle of three steps: (i) agents observing their neighbourhood’s aggregated usage over the period since the last decision time-step, (ii) agents making decisions according to the rules of the schema, and (iii) agents updating their inner state and behaviour to create a new predicted load profile.
- The agents observe but do not influence each others decisions directly. A simple controller in each dwelling can then use this information to make decisions which affect the output of the model.

The system is then simulated for a period of one week (this study does not consider seasonal variation of electricity demand hence, for simplicity, a single week is considered throughout this work). The choice of these time-frames will not significantly affect the results, given that longer simulations would be a repetition of the basic daily cycles and hence not affect the nature of the decisions, which is the central aspect of this investigation.

The ABM has a stochastic element in its individual runs in order to model a variety of household typologies and different behaviour patterns, hence multiple runs allow the variability in the model to be captured^83^. Initial trials of up to 30, 50 and 150 runs of the ABM demonstrated that data variability plateaued at around 30 runs. Hence the model was run 30 times for each scenario and the outcomes of these ensemble runs were averaged.

### Model set-up and implementation

As thermal loads are more constrained in a large number of buildings the initial model excludes these and only looks at smaller, more shiftable loads. Subsequently, thermal loads are included in a more time-constrained way, assuming that modern construction techniques will make such time-constrained load shifting more feasible in the future.

#### Model initialisation

The ABM for peak coordination and reduction was implemented using the open source *RePast Simphony* agent based modelling environment written in the Java programming language^84^. The system initialisation (Fig. 2a) includes:

(i) internal properties (described below);
(ii) network connections (Fig. 2b)—described in Section “Network topologies” and listed in Table 1;
(iii) external factors: system parameters including total system size, threshold ($\tau$) for action, time-window for shifting of appliances and each agent’s neighbourhood’s maximum demand level (see Section “Model set-up and implementation”).

Once the input data have been provided, the internal properties of the “dwelling” agents in the network are initialised. Each dwelling is assigned a set of loads representing appliances according to appliance ownership rates defined in^52^. For example, if the ownership rate for the appliance *A* is 80% then 80% of the dwellings will be selected randomly and the appliance *A* added to the list of appliances they own. To guarantee variable and realistic appliance usage schedules, initial appliance time schedules are generated from a truncated normal distribution, based on type of occupancy discussed in detail in Section “Model set-up and implementation”. Afterwards, initial consumption patterns for appliances are generated for each dwelling agent, based on occupancy types and statistics such as occupant activity/inactivity times. The last step of the initialisation is to define the network of interactions between dwellings, based on the types given in Section “Model set-up and implementation”.

#### Peak coordination algorithm

After setting the model parameters, initial load demands (Section “Model set-up and implementation” and Fig. 2a) and network topology (Section “Network topologies” and Fig. 2b) the peak coordination algorithm is initiated, based on the actions set out in Section “A simple schema for load coordination”, and shown in detail in pseudo-code in Algorithm 1. The aim of the algorithm is to determine the action to be taken at the next time-step *t*, based on the previous state at the preceding 15-min interval $t-1$.

For each time-step *t*, the model updates the properties and behaviour of every agent and obtains the sum of each agent’s neighbours’ electricity demand at time $t-1$. The neighbourhood peak load, $l_{max}$ is modelled by summing the peak loads that would occur within an agent’s *closed* neighbourhood (that of itself *and* its network neighbours) over an arbitrarily chosen one week time-scale without the peak coordination algorithm. This simulates the situation where a period from the previous system history would be used to estimate $l_{max}$. A threshold is then calculated by multiplying $l_{max}$ by a scaling factor $\alpha$. The parameter $\tau = \alpha \times l_{max}$ subsequently acts as a *load redistribution limit* and controls the total amount of load that can be shifted in each time-step. The case of $\alpha =0$ corresponds to no load-shifting and is therefore reverts to the baseline-case, whereas $\alpha =1$ allows agents the potential to simultaneously shift all load to the same time and hence cause a new peak where there was once a dip in demand.

Next, the electricity consumption of each dwelling agent and its network neighbours at time $t-1$ is compared with $\tau$ and one of two actions is taken, as follows. If electricity consumption in the closed neighbourhood is greater than or equal to $\tau$, the decision to decrease electricity demand at time step *t* will be made and a load that can be shifted will be identified from the appliance list. The load is then shifted to a *demand pool*, to be rescheduled within the defined shifting time window *N*. Otherwise, if the electricity consumption of the dwelling is below $\tau$ the decision to increase electricity demand at time step *t* will be made and an appliance-load within the demand pool will be identified. The electricity demand for the agent will then be updated for that time step. The simulation then outputs the computed electricity loads for each dwelling in 15 min intervals over one week. This process is then repeated for each 15-min interval for the whole computed week, giving a total of 672 steps per run.

#### Algorithm details

The algorithm below illustrates the sequence of steps described above. Defining the set of all dwelling agent nodes $D=\{d_1,d_2,\ldots ,d_n\}$, the undirected network of agents is denoted as a graph *G*(*D*, *C*), connecting nodes *D* via links given by $C = \{(d_i,d_j)\}$ where $1 \le [i, j] \le n$ and $i\ne j$. The neighbourhood of agent $d_i$ is given as:

$$\begin{aligned} N(d_i) =\{d_j \mid (d_i, d_j) \in C\}. \end{aligned}$$

Further, the *closed* neighbourhood of $d_i$ is defined as the set containing both $d_i$ *and* its neighbourhood $N(d_i)$, given by the union $N[d_i] = d_i \cup N(d_i)$. The electricity consumption of an agent $d_i$ at time step *t* is denoted $e(d_i, t)$, so the electricity consumption of agent $d_i$’s *closed* neighbourhood at time *t* is thus given by:

$$\begin{aligned} E_{N[d_i]}(t) = \sum _{d_j \in N[d_i]}e(d_j, t). \end{aligned}$$

Similarly, $\hat{e}(d_i)$ denotes the *sequence* of all demands for every 15 min interval in $1\le t\le 672$ for agent $d_i$ over the whole week, and the sequence of electricity consumption values for $d_i$’s closed neighbourhood is the sum over this and denoted $\hat{E}_{N[d_i]}$. Hence the peak electricity consumption of agent $d_i$’s *closed* neighbourhood is defined as:

$$\begin{aligned} \textrm{Peak}_{N[d_i]}=\max _{1\le t\le 672}\hat{E}_{N[d_i]}. \end{aligned}$$

The pseudo-code in Algorithm 1 below shows workflow of the ABM:

**Algorithm 1:**
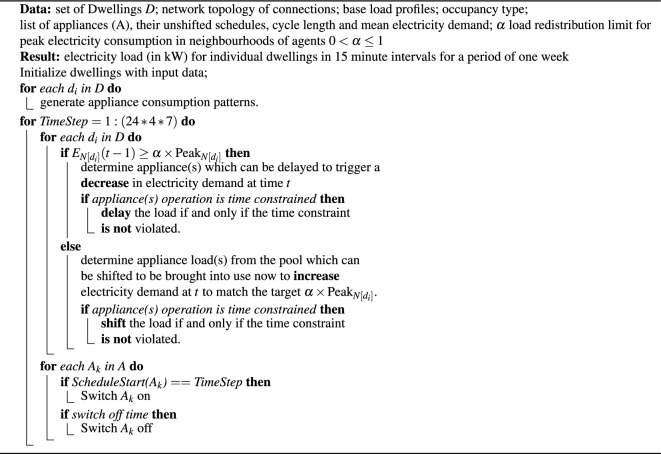
Peak coordination algorithm

#### Network initialisation

The following network topologies were generated using Python library NetworkX^85^ and Java library jGraphT^86^: partition networks with probability of links within communities $p_1=1$ and probability of links between communities $p_2=0$—representing disconnected neighbourhoods which are each internally fully connected; small world networks with a rewiring probability of zero (WS($p=0$))—i.e., simple ring lattices with various degrees representing a system of connected neighbourhoods; small world networks with 100% rewiring probability (WS($p=1$))—i.e., random networks with no community structure; configuration model networks with each node having pre-determined constant degree *d* (CM($d=2$), CM($d=2$) and CM($d=8$))—giving random networks with fixed (rather than distributed) degrees. Table 1 shows detailed statistics of the networks. Some partition networks have 99 nodes to generate network topologies with comparable average degrees, and no loss of generality.

**Table 1:** Networks statistics, showing different network generation models and parameters: defined degree *d* for the configuration model (CM); rewiring probability *p* for the small world (WS) networks; and number of communities for the Partition model. Also shown are some of the resulting measured topological features: average degree (number of links) and clustering coefficient (degree of *co-connectivity*).

Network topology	Average degree	Edges	Communities	Clustering coefficient
Partition	1	50	50	0
Partition	2	99	33	1
WS($p=0$)	2	100	–	0
WS($p=1$)	2	100	–	0
CM($d=2$)	2	100	–	0
Partition	4	200	20	1
WS($p=0$)	4	200	–	0.5
WS($p=1$)	4.06	201	–	0.03
CM($d=4$)	4	200	–	0.02
Partition	8	396	11	1
WS($p=0$)	8	400	–	0.64
WS($p=1$)	8.06	400	–	0.06
CM($d=8$)	8	400	–	0.05
Partition	10	495	9	1
WS($p=0$)	10	500	–	0.66
WS($p=1$)	10	500	–	0.09
CM($d=10$)	10	500	–	0.08

#### Base load profiles

**Figure 3:**
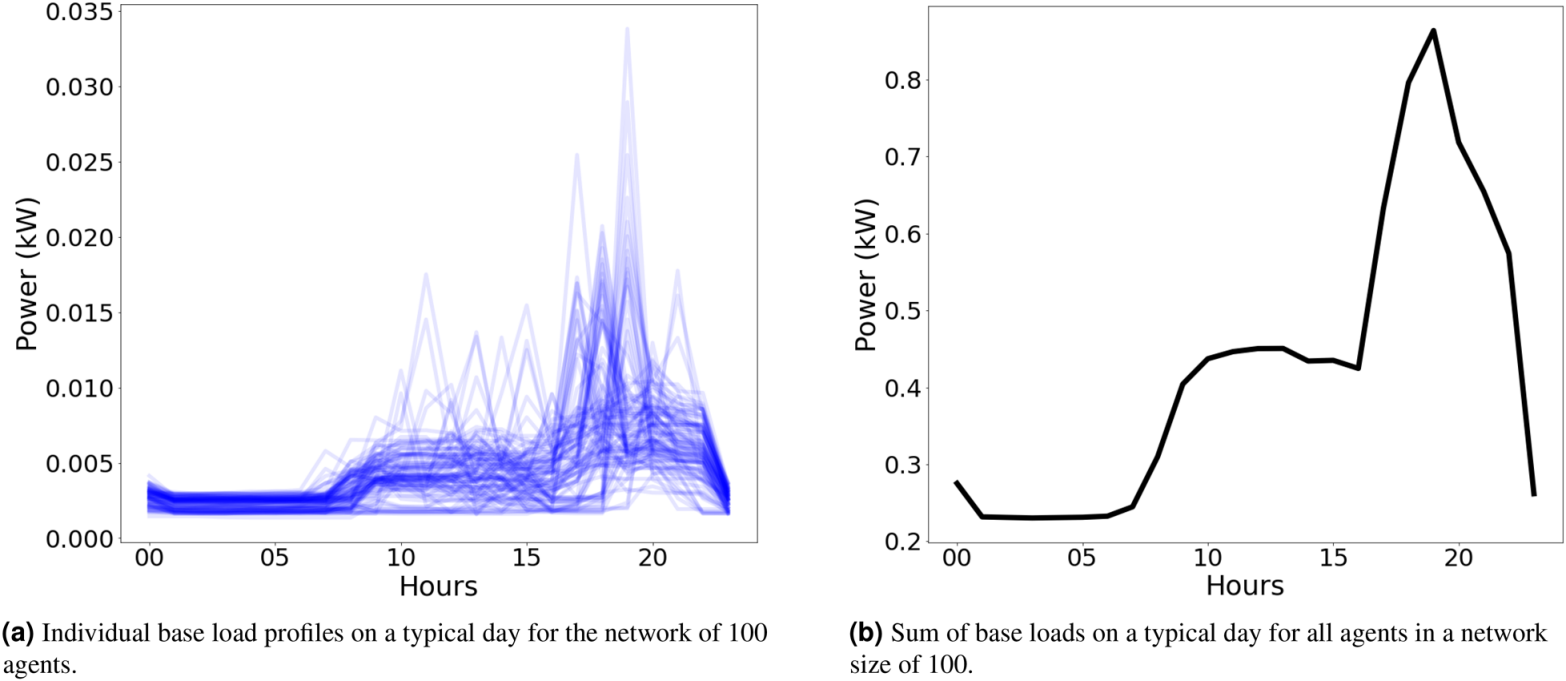
Example base load profiles over a single weekday for (a) 100 individual agents and (b) total base load for all agents.

Base loads (often called *static loads*) represent uncontrollable energy demand. These loads cannot be influenced by control systems and have no inherent flexibility—being either always on, or a collection of on-demand loads. Each agent in the network was initialised with an individual base load electricity demand. The generation of profiles for networks of 100 buildings was done using the “Artificial Load Profile Generator for DSM” (ALPG) tool^52^. This open source tool generates realistic, high resolution load profiles through simulation of occupant behaviour, validated against measurements obtained in a field-test^52^. The base load profiles are illustrated in Figure 3.

#### Occupancy types and schedules

There exist a wide variety of domestic occupancy types depending on the number of people in a household, their ages, employment status etc.,^87^. For simplicity, just two were considered here: “employed”and “unemployed” (in a 70:30 distribution ratio) as it provides the two extremes of “intermittent” and “permanent” occupancy, respectively. An advantage of this simplification is that it reflects the profiles used in the ALPG tool noted above. The main difference between the two occupancy types is that a dwelling with “employed” occupants will initially be scheduled to only use appliances in the mornings or in the evenings, whereas appliances are scheduled randomly throughout the day for “unemployed” occupants.

#### Model constraints

Since the focus of this work was to investigate the impact of the network topology and average number of neighbours on the peak coordination schema it was assumed that all shiftable appliance loads can be shifted by the peak coordination algorithm (see Section “Introduction”), so factors such as appliance priority or appliance run-time factor—the ratio of time for which a particular appliance was in the running state during the previous time slot—are not included in the current scheme. Initially only highly shiftable appliance loads were modelled, in order to investigate the effectiveness of the algorithm in terms of its most effectiveness in the absence of thermal loads. However, given that thermal loads are usually the single largest load type, it is important to understand the overall effectiveness including the larger thermal loads, which were included in a second iteration of the model, described below.

#### Thermal loads

Unlike other loads which are considered to be largely unconstrained, thermal loads usually operate in discrete intervals related to the need for the load—arising from a combination of weather and lifestyle. In the UK, for example, a pattern of heating once in the morning and once in the evening is common, with the typical length of each heating event varying between 2–3 h^88^. For convenience and simplicity, the typical heating pattern in the UK is used as an embodiment typical of thermal loads and it is assumed that (i) all the dwellings in the network have a heating operation twice a day (ii) the length of each heating event is fixed and equal to 2.5 h and (iii) the typical power rating for heating load is 12 kW, a sufficiently large capacity for most common heating loads, including heat pumps^89,90^.

To ensure variability and simulate the well-known “demand diversity factor”, a heating schedule is generated for each dwelling in the network by randomly sampling within fixed intervals in morning (05:00–08:00) and in the evening (17:00–19:00).

The time shifting window for heating loads will, in practice, be considerably smaller than for non-thermal loads due to the lower flexibility in how much they can be shifted. For example, there would be little use in supplying heat to a home six hours after it is usually needed. However, heating demand can be considered predictably stable during the summer in cooling dominated, and winter in heating dominated, climates, allowing thermal loads to be brought forward in addition to being delayed. Hence, the time shifting windows for the heating loads is constrained to a narrower band of either one or two hours either side of “pre-scheduled” demand. In other words, the total available window for heating operation is increased by either one hour either side of the scheduled demand or two hours either side. While the need to know heating or cooling schedules increases the information needed to implement the schema, it is a quantity that can be readily obtained from a modern domestic controller. Moreover, as building standards improve to reduce conductive heat transfer, thermal loads will become smaller and more flexible. The heating loads are modelled here as electrically driven, not only for convenience but to align with the phasing out of gas boilers and move within the industry to electrical sources of heat provision.

**Figure 4:**
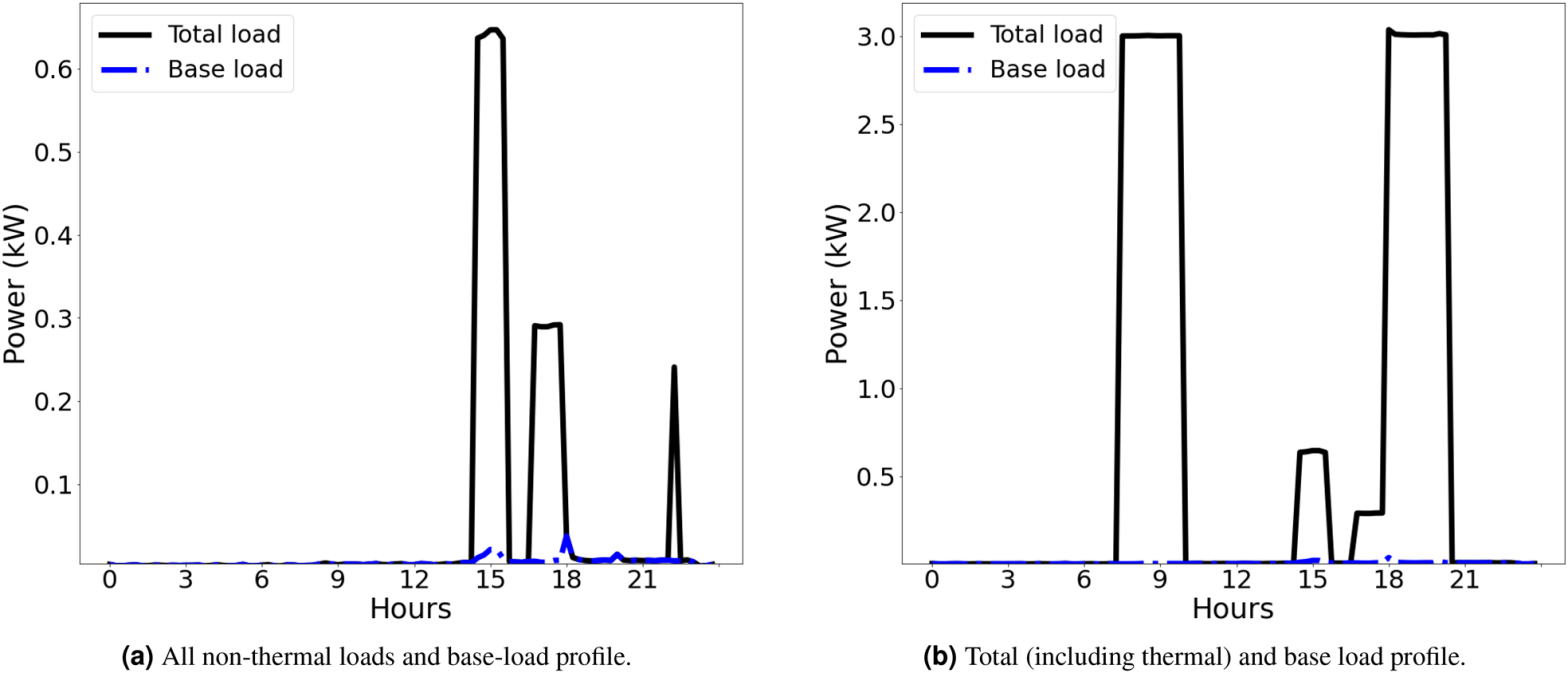
Example load profiles for a single, randomly selected, dwelling on a typical weekday. The large 3 kW peaks are from the heating system, whereas the smaller peaks are from other appliances, per Fig. 3.

Figure 4 illustrates the base and total load for a single, randomly selected, dwelling in the simulation. Compared to the load produced by the simple ABM model presented in Section “Methodology” with no heating system included (Fig. 3) the total load when a heating system is included is significantly higher, as expected.

### Experimental set-up

To partially validate the numerical results as well as demonstrate the practicality of the load-shifting schema, an experimental set-up was constructed at the Building Research Park (BRP), located near Swindon in the UK and operated by the University of Bath.

**Figure 5:**
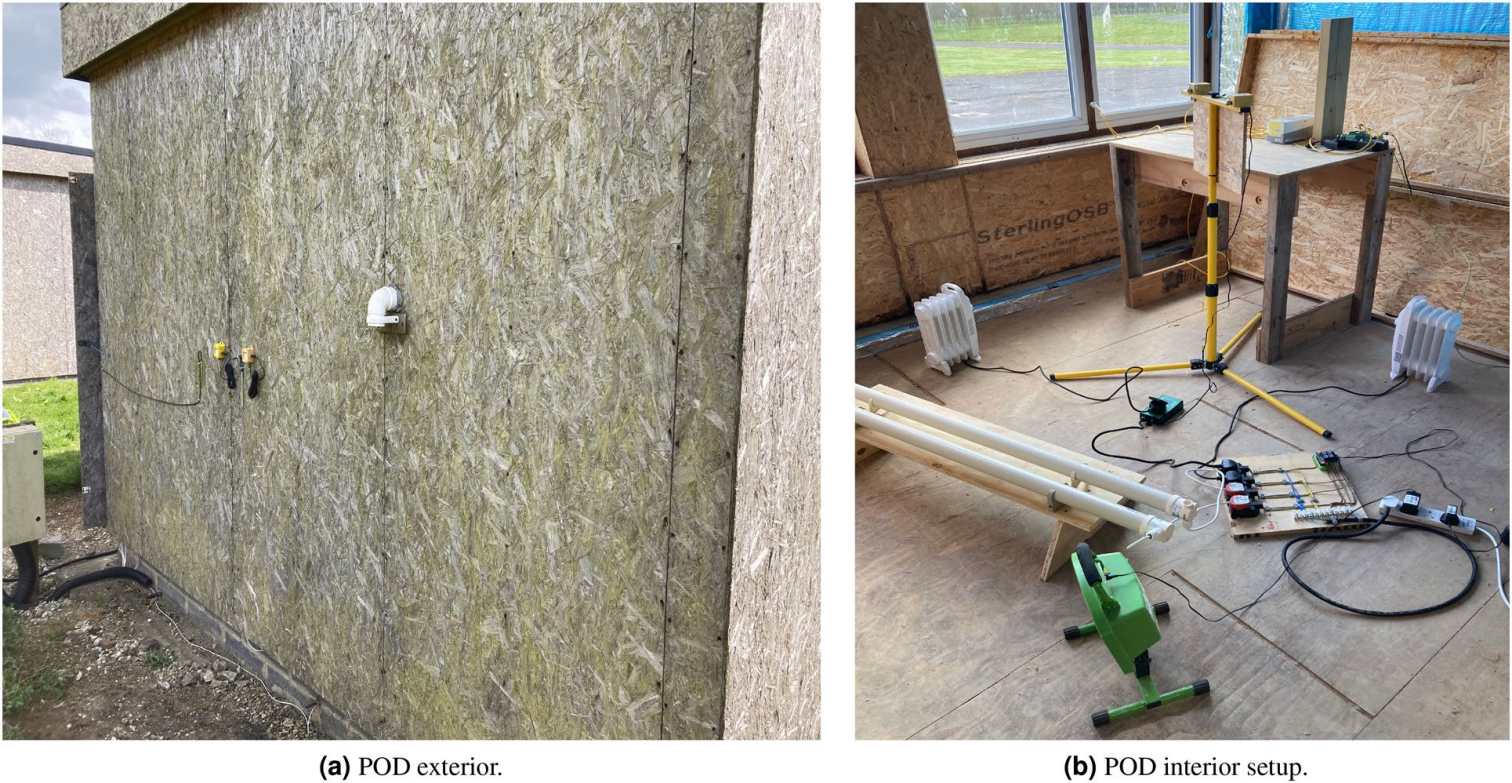
Set-up for the demonstration experiment, showing (**a**) the exterior of one POD and (**b**) the model appliance loads being controlled by the micro-controller and relays.

This consisted of nine well-insulated constructions (called PODs) in an isolated location, in to which a set of example loads were installed, each controlled by a raspberry-pi based controller and relay board (Fig. 5). The microcomputer in each POD was programmed with the load coordinating schema, equivalent to the ABM in the numerical simulations. Each of the nine PODs was associated with a neighbourhood group of three, thus creating a model system of three groups.

Data exchange and control of the experiment was performed by wireless communication with Google Sheets API from the python scripts running on the raspberry-pi micro-controllers. An initial schedule for the loads was set up for each POD, with loads representing some common household loads, as shown in Table 2. Note that typical domestic loads are scaled to the size of the PODs and furthermore are only example loads for the purpose of demonstrating the effectiveness of the schema.

**Table 2:** Example schedule for an experimental dwelling prior to load shifting, representing a baseline control case.

Demand	Time on	Time off	Load (W)
Heating	06:30:00	08:30:00	650
Heating	12:00:00	14:00:00	650
Heating	18:30:00	23:00:00	650
Hot water	06:00:00	08:00:00	650
Hot water	18:00:00	22:00:00	650
Washing machine	12:30:00	14:00:00	240
Dryer	19:00:00	20:00:00	300
Baseload	00:00:00	23:59:59	180

At each time interval of 15 min the PODs observed the aggregated load of their group, then acted to adjust their own schedule. The actions were either to either (a) delay some load into a “demand pool” if the group was above a pre-calculate average or (b) bring a delayed load (or available thermal load) forward if it was both (i) needed to fill a shortfall and (ii) available in the demand pool. The algorithm was permitted to shift the “heating” loads up to one hour forwards or 1 h backwards in time, in intervals of 15 min, and could delay each of the other loads by up to 6 h maximum. Once a load had been delayed, it could subsequently be reinitialised at any point between its originally scheduled start time and the delayed time if it was needed to fill a demand gap.

## Results

### Overview

The results below demonstrate the potential effectiveness of the load-coordination schema implemented at the building (node) level—which is simple, requires little data exchange or human intervention and relies on only simple rules and minimal interaction between buildings. The results in Section “Simulation scenarios”–“Scenario 2: the impact of network topology and node degree” exclude thermal loads, before moving on to show how their inclusion affects the effectiveness of the schema in Section “Inclusion of thermal loads”. Since the interaction of buildings will occur via the information flows modelled as links of the interaction network connecting them, a range of network topologies were examined, with a view to investigate their impact on any resultant peak load reduction. Alongside this, the key model parameters were investigated to find an optimum set of values, in terms of the minimum *peakiness*—measured in terms of the root-mean-square-error (RMSE) deviation from a totally flat, average load.

### Simulation scenarios

The parametric investigation of the model is separated into two scenarios (shown in Table 3 in Section “Model set-up and implementation”), with Scenario 1 having a fixed number of directly linked neighbours (average node degree) but variable time windows for demand shifting and Scenario 2 having a range of degrees but fixed time shifting window.

Two scenarios were implemented in the ABM model to analyse the system parametrically, one with each node having a fixed number of directly linked neighbours (average node degree) but variable time windows for demand shifting and the other with a range of degrees but fixed time shifting window, as shown in Table 3. Shorter windows were also tested but did not show any significant reduction of peaks from that when no schema was applied.

**Table 3:** ABM simulation scenarios. Network topologies varied between: partition, ring lattice (WS($p=0$)), random using the small world scheme (WS($p=1$)) and the configuration model with fixed node degree (see Table 1 for details). The average degree is the mean number of directly connected neighbours of each node in the network. $\alpha$ is the load redistribution limit (Section “Model set-up and implementation”) increased at intervals of 0.05 over the indicated range. Time window is the maximum interval of time that a load can be shifted within, with the actual length of shift being randomly determined. Hence, Scenario 1 consists of $4\times 1\times 21\times 3 =252$ simulations, and Scenario 2 consists of $4\times 4\times 21\times 1 = 336$ simulations.

Scenario	Network topology	Average degree	Load redistribution limit interval	Time window
Scenario 1	Four types	4 (fixed)	$0\le \alpha \le 1$	3 h, 6 h, 12 h
Scenario 2	Four types	2, 4, 8, 10	$0\le \alpha \le 1$	3 h (fixed)

#### Scenario 1: impact of time shifting window

**Figure 6:**
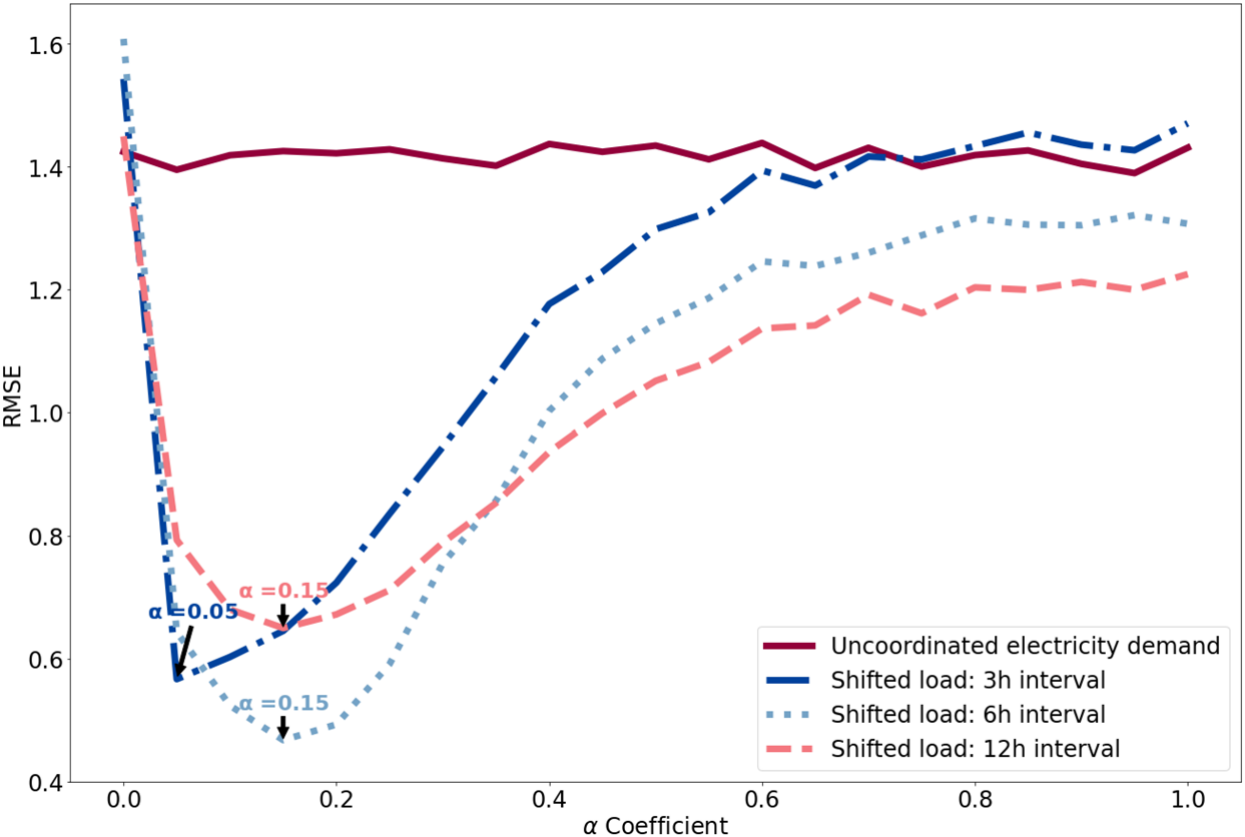
Results for Scenario 1, showing the impact of time shifting window using our schema on peak-load reduction. The RMSE values show the deviation from a flat demand profile, with zero being peak-free. Partition networks were used, consisting of a hundred dwellings with neighbourhoods of size five (i.e., node degree $d=4$). The minima of the three shifted curves are indicated by arrows at $\alpha =0.050$, $\alpha =0.15$ and $\alpha =0.15$ for a shifting window of maximum of 3 h, 6 h and 12 h, respectively. Results obtained using other network topologies appear almost identical.

Detailed analysis of the model outputs shows that for all network topologies, the peak reduction schema is most effective (i.e., achieves the most peak load flattening) when the load redistribution limit $\alpha \approx 15\%$ and the time shifting window is up to 6 h. For example, in the network with partition topology shown in Fig. 6 the lowest RMSE of 0.46 is recorded for when the time shifting window is 6 h. Time shifting windows of 3 and 12 h lead to higher RMSE values of 0.57 and 0.65 respectively. The RMSE values shown in Fig. 6 illustrate the dependence of the effectiveness of the peak reduction schema on both the time shifting window and load redistribution limit $\alpha$. Scenarios where $\alpha =1$ or $\alpha =0$ are where all agents in a neighbourhood end up following the same tactic, which results in peak demand shifting from one point of time to another or remaining the same with no flattening achieved. This is equivalent to a peak reduction strategy that operates solely at the individual building level.

Importantly, while clear minima in RMSE are identified at a particular load distribution limit, the width of a given curve indicates the robustness of the different configurations. That is, the less steep the trough of a curve, the more robust the given configuration to produce lower peak demand.

#### Scenario 2: the impact of network topology and node degree

**Figure 7:**
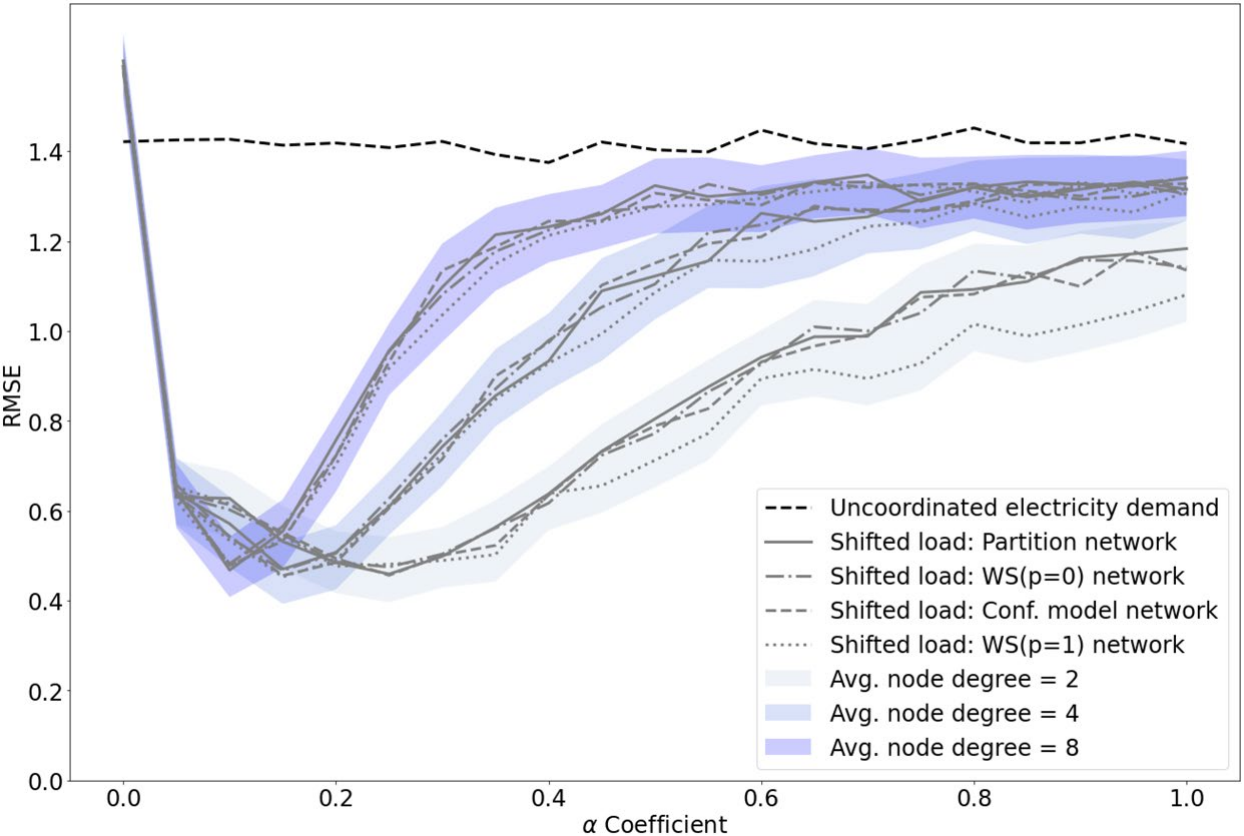
Results for Scenario 2, showing the impact of network topology on peak load reduction. Data are shown for a six hour time shifting window as this was the result with the lowest RMSE in Scenario 1. Each coloured series shows the mean (lines) and range (shading) of the shifted demand for four network topologies: partition, ring lattice (WS($p=0$)), random configuration model and random (WS($p=1$)). FHWM bandwidth for node degrees 2, 4 and 8 are 0.58, 0.4 and 0.24 respectively. FHWM bandwidth for node degrees 8 and 10 are virtually identical and hence only 8 is shown.

The results for Scenario 2 reveal that, surprisingly, network topology does not significantly influence the peak reduction behaviour of the implemented peak coordination schema. This can be seen in Fig. 7 by the simulation results for very different network topologies having strikingly similar RMSE values over the range of $\alpha$ values. The slight differences between random (WS($p=1$)) and other network topologies are likely due to the variation in node degrees inherent in such random networks.

The most interesting result here is the impact of average node-degree (see Section “Network topologies”), i.e., number of neighbours, on peak demand reduction. While all node-degrees share similar RMSE minima, their gradients increase more steeply with higher node-degree as $\alpha$ increases. The width of our curves can be compared using the well-known full width half maximum (FWHM) bandwidth, which reveal that, for all network topologies, RMSE curves for average node degree 2 are approximately twice the width of those with average degrees 8 and 10. However, the FWHM widths for networks with average node degrees 8 and 10 do not show any significant differences. This indicates that our peak coordination schema is most robust over a broad range of the load redistribution limit $\alpha$ for networks with node degrees 2–4, but its effectiveness is limited to a very narrow range of $\alpha$ values with higher node degrees.

The overall impact of these results on peak demand reduction is significant. An analysis of all implemented scenarios shows that the largest reduction of peaks in electricity consumption (average 59%, standard deviation 13% and maximum of 73%) can be achieved in the networks of node degree 4 and with $\alpha$ values ranging between 5 and 25%. The reduction of peaks regardless of the choice of $\alpha$, on average, is 31% with standard deviation 21%. While this striking difference highlights the utility of choosing an optimal value for the load redistribution limit $\alpha$, it also demonstrates that significant reductions can be robustly obtained across a wide range of chosen values for $\alpha$.

### Inclusion of thermal loads

For the extended ABM model, the scenario group with the best parameters from the initial model in Sections “Scenario 1: Impact of time shifting window” and Scenario 2: The impact of network topology and node degree was used, which guaranteed the greatest peak demand flattening results (Table 4). This has a time window of 6 h and network average degree of four neighbourhood members. The extended model is less able to achieve peak flattening due to the significantly larger loads that are also more highly constrained in how much they can be shifted (either one or two hours either side of the scheduled demand, as mentioned in Section “Model set-up and implementation”). Most of the flattening comes from using the most optimal scenario group from the non-thermal case.

**Table 4:** Scenario group parameters for extended ABM simulations.

Network topology	Load redistribution limit interval	Time window (shiftable loads)	Time window (heating loads)
CM($d=4$) (See Table 1)	$0\le \alpha \le 1$	6 h	2 h (1 + 1),
			4 h (2 + 2)

**Figure 8:**
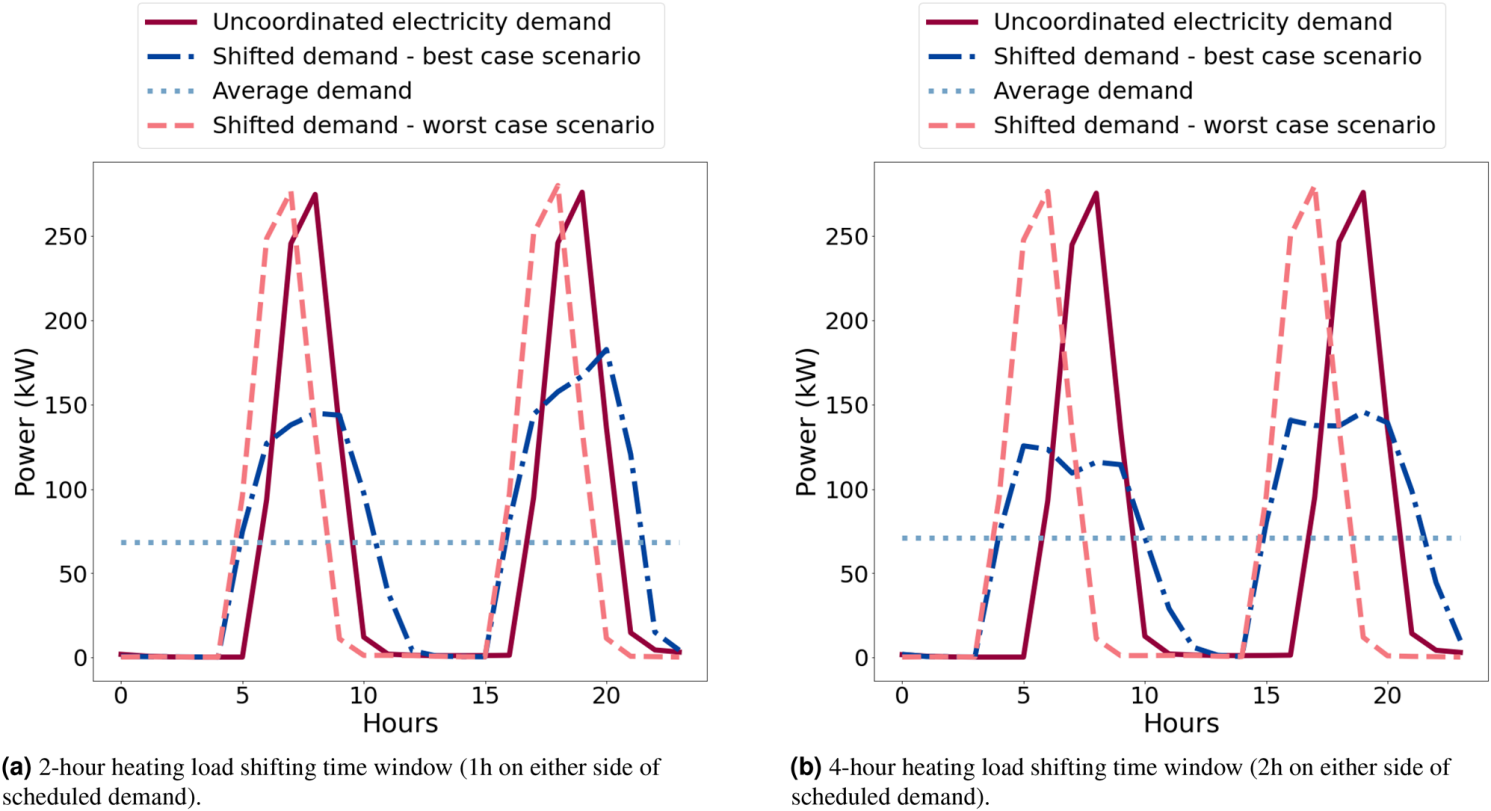
Hourly load profile of a network of hundred dwellings with node degree $d = 3$. Data show coordinated demand for two-hour and four-hour heating load shifting windows (best and worst cases) against constant average and uncoordinated demand.

The extended model achieves the greatest peak electricity demand flattening when the load redistribution limit $\alpha \approx 10\%$ and the time shifting window for heating loads is up to four hours. Figure 8 illustrates the typical load profile of a network with three neighbours with uncoordinated and coordinated loads. These results show that while a two-hour time window of shifting heating operation results in a maximum 44% of peak flattening (mean 21%, s.d. 14%), a four-hour time window in a maximum 61% reduction (mean 33%, s.d. 23%), consistent with the idea that greater flexibility would result in greater flattening. Note that the minimum reduction for both scenarios is zero. Furthermore, the analysis of maximum ramp rates (kW per half-hour) shows that reductions of 31% and 29% can be achieved for four-hour and two-hour time windows correspondingly. These results, though lower than for non-thermal loads, show substantial reductions and are consistent with the earlier findings.

### Analysis of heating operation schedules

**Figure 9:**
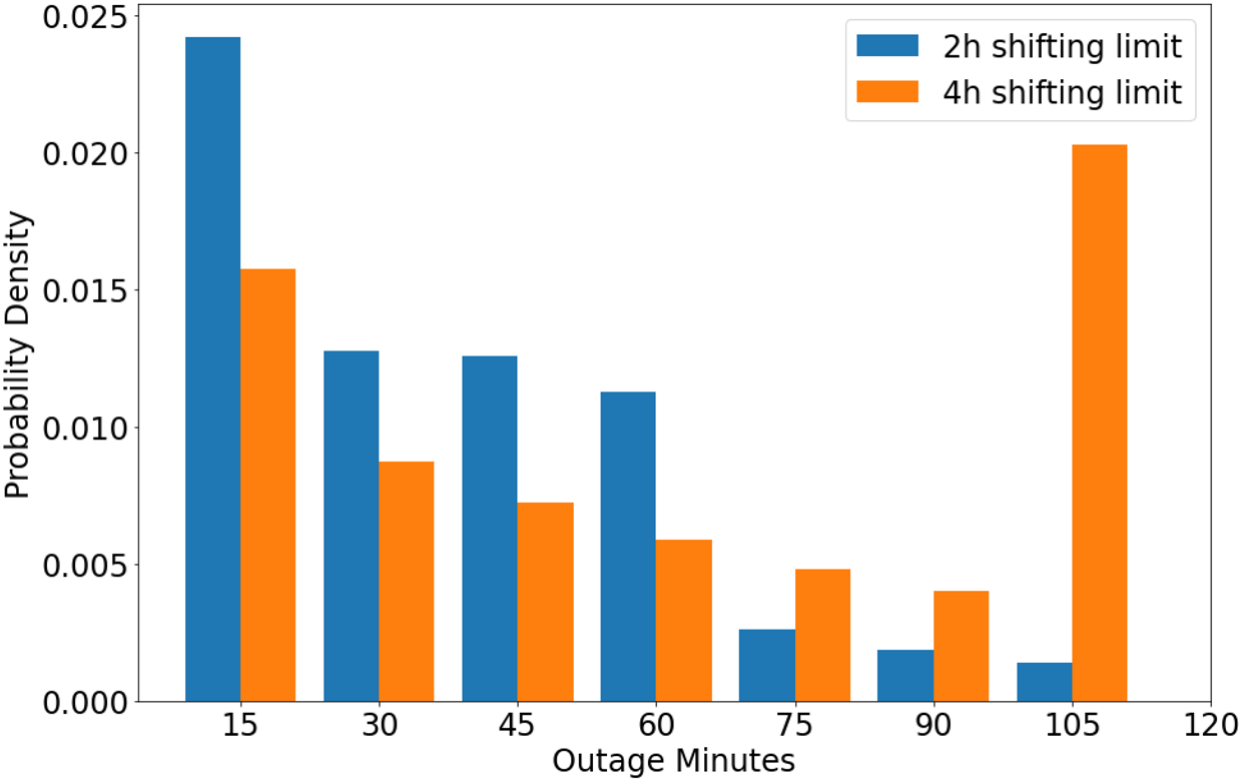
Normalised distribution of run-length of heating operation outage hours for 2-h and 4-h heating load shifting time window.

Since thermal energy demand drives indoor comfort and the extended model will unpredictably interrupt the operation of the heating or cooling system, it is important to investigate the extent of disruption to the heating schedule that would result. This was investigated by analysing the distribution of run-lengths of heating operation outage hours for each dwelling in the network. That is, how long, on average and maximum, does a typical home experience an interruption in heating during the morning or evening period? The normalised distribution of run-lengths of outages for both the two-hour and four-hour time window scenarios in Fig. 9 suggests that the most common heating outage length is 15 min for the two-hour window, while it is 105 min for the 4-h window. This is due to the fact that the system is able to advantageously use the larger window of 4 h to simply shift longer heating periods, whereas it is “forced” to break this schedule up into smaller chunks in the two-hour window. Longer breaks are also evident in the two-hour window, but are significantly less likely to occur. Further analysis shows that, on average, a single building is expected to have two outage events per day for the 4-h shifting window, with an average event length of 66 min, standard deviation of 42 min and total outage length of 162 min. For the 2-h shifting window, we observe an average of three outages per day with an average event length of 38 min, standard deviation of 23 min and a total outage length of 120 min.

### Experimental demonstration

**Figure 10:**
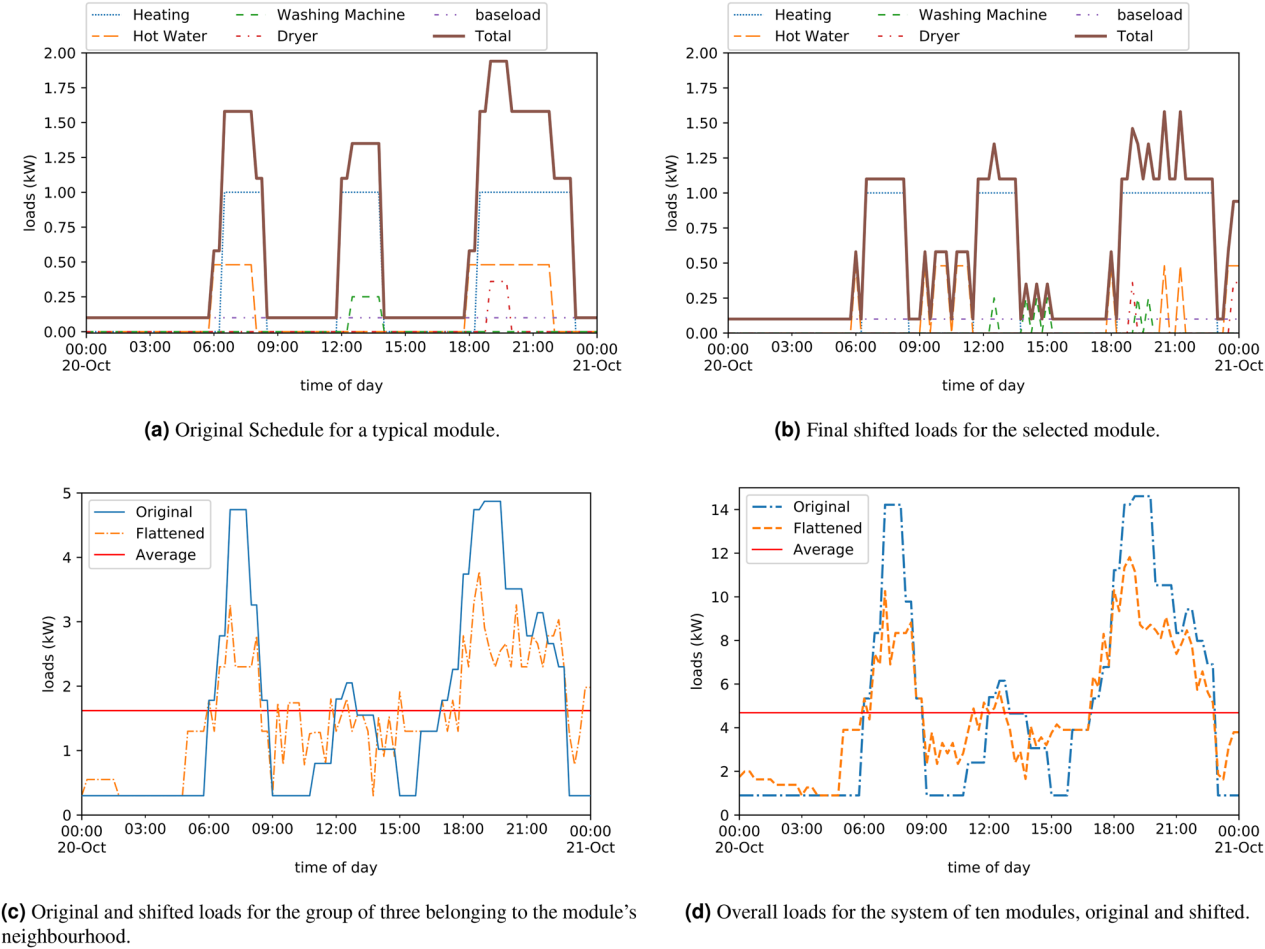
Experimental demonstration results, showing the shifted and unshifted loads for a representative example POD.

The demonstration was run over several weeks with different configurations in order to troubleshoot and verify that the scheme could be effective in a realistic setting. Figure 10 shows the schedules over a typical day. The original schedule is shown in Fig. 10a before any rescheduling took place. The RMSE value was 6.5 kW, baselining the peakiness of the projected load profile. At the end of the day, when all rescheduling had taken place the shifted load profiles were noticably flatter, as shown in Fig. 10b, with a RMSE of 4.8 kW—a reduction of around 25% for this one arbitrarily chosen POD. This is despite the thermal loads being hardly shifted at all in the example shown. However, to relieve load on the grid, the main aim is to flatten load at the group or system level. The original unshifted and flattened aggregated load for the group of three associated with this POD is shown in Fig. 10c. The original RMSE was 14.8 kW and for the flattened load 8.7 kW, which is over 40% reduced in peakiness. Finally the system of nine PODs aggregated load is shown in Fig. 10d, with an original RMSE of 44.5 kW and a flattened load RMSE of 27.9 kW, again reduced by just under 40%. The similarity of these two numbers is unsurprising given the numerical results showing that the main effectiveness in load flattening is amongst small groups and that larger systems are a simple linear scaling of this basic result.

## Discussion

The main results arising from this work are that interactions between only small numbers of buildings are needed to achieve significant peak-load reductions, that these are mostly independent of network topology and that they are robust over a wide range of other key parameters. These new insights are also interesting from the perspective of complexity and network science, as they reinforce findings in other application areas that the theoretically optimal size for coordination in networks may be surprisingly small, much like in models of flocks of birds and other natural systems. The greatest peak demand flattening occurs when the time shifting window is around six hours, the network degree (i.e., number of neighbours) is four buildings and the load redistribution limit $\alpha$ is between 10 and 25% of the neighbourhood’s peak load. When $\alpha =1$ or $\alpha =0$ no peak reduction is seen in the network. This is similar to game theoretic approaches—used to study the formation of networks—which conclude that, if agents can observe each other’s actions and outcomes over time and all agents have the same preferences and face the same form of uncertainty, then they develop similar payoffs over time^91^. However, it can be seen here that even a poor choice of $\alpha$, on average, results in a 31% reduction in peak demand. Hence, the theoretical range of possible reductions predicted by our approach (31–73%) are far greater than even the predicted range of reductions in the DSM literature of between 13 and 50%.

**Figure 11:**
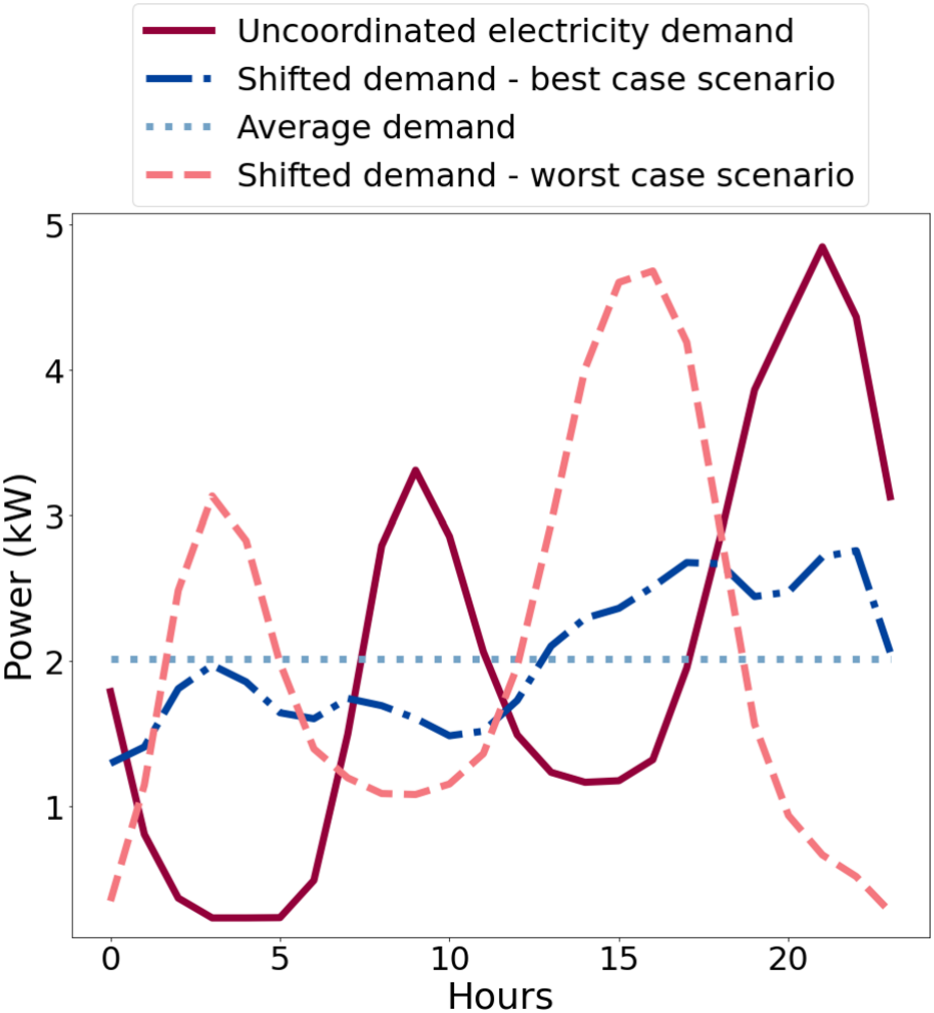
Daily typical total load profiles of a network with just three connected neighbours, comparing coordinated demand (best and worst cases) against constant average and uncoordinated demand.

Figure 11 illustrates the typical load profile of a network with just three neighbours with uncoordinated (i.e., occurring near-simultaneously) and coordinated loads, the latter for both highly optimised (best case) and non-optimised (worst case) parameters. The effect on ramp rates is also dramatic. That is, for the best case peak reduction scenario (i.e., $\alpha =0.15$) the maximum ramp rate (kW/half hour) of schema-coordinated load can be reduced by 65% of the maximum ramp rate of the original, uncoordinated, load. In contrast for the worst case scenario when $\alpha =0.95$, the maximum ramp rate of schema-coordinated load is 8% higher than the maximum ramp rate of the original uncoordinated load.

Strikingly, the results demonstrate that network topology does not significantly influence peak flattening. This suggests that it is the simple presence of connections between dwellings that is important, rather than the manner of connection. However, there is a limit to the utility of the number of connections, or average degree of the network. Not only does increasing the average degree not improve the effectiveness of the peak coordination strategy, but it also limits the effective range of $\alpha$ to a very small window. This is analogous to the social behaviour of animals where it has been observed that, due to homogeneous interaction, animal social contact networks are not scale-free (i.e., node degrees do not follow power-law degree distribution)^92^. A second notable similarity with models of biological systems—such as flocking birds—is that the optimal number of neighbours is small. For example, birds are known to interact with a small number (6–7) of neighbours to form a flock (^46,43^).

The impact of the window within which behaviours can be shifted is observed to be optimal at up to six hours (RMSE $=0.46$), though substantial reductions in peak demand can also be observed at the other intervals, with the 12 hour window being the “worst”. In this case an RMSE of 0.65 is obtained, i.e., 19% worse than the six hour window, but still 54% better than with no coordination (RMSE $=1.42$). It is noteworthy that the time window only specifies the range within which an energy consuming action can be deferred. In our modelling, agents randomly distribute loads within this window, which is consistent with the behaviour of an unmediated (i.e., automatic) controller. To what extent such behaviour can be expected from human-mediated action, were such mediation deemed useful, remains to be seen.

Given that thermal loads, such as those from a heating or cooling system, tend to be large in absolute terms and the key driver of peak loads, it is pertinent to ask whether such loads can be deferred to help balance loads. After all, the impulse to use heating and cooling is strongly dependent on external weather conditions, and there may be little flexibility in the timing of these loads. However, unlike some appliances, such as some washing machines whose individual cycles may be hard to interrupt once started, heating and cooling systems are fundamentally *interruptible*. Hence, it is possible, in principle, for a heating or cooling system to temporarily interrupt operation, with the possibility of restarting in the next 15 min interval. This is entirely within the remit of the schema propose here since there is no decision memory—the system makes decisions independent of those made in previous intervals. Finer time-scale dynamics were excluded in this initial study, where thermal loads are regulated within set limits by switching on or off, as our loads are either on or off during the entire 15-min interval to represent worst and best cases, respectively. However, distributed on-off cycling of loads is also a possibility for flattening out the loads.

**Figure 12:**
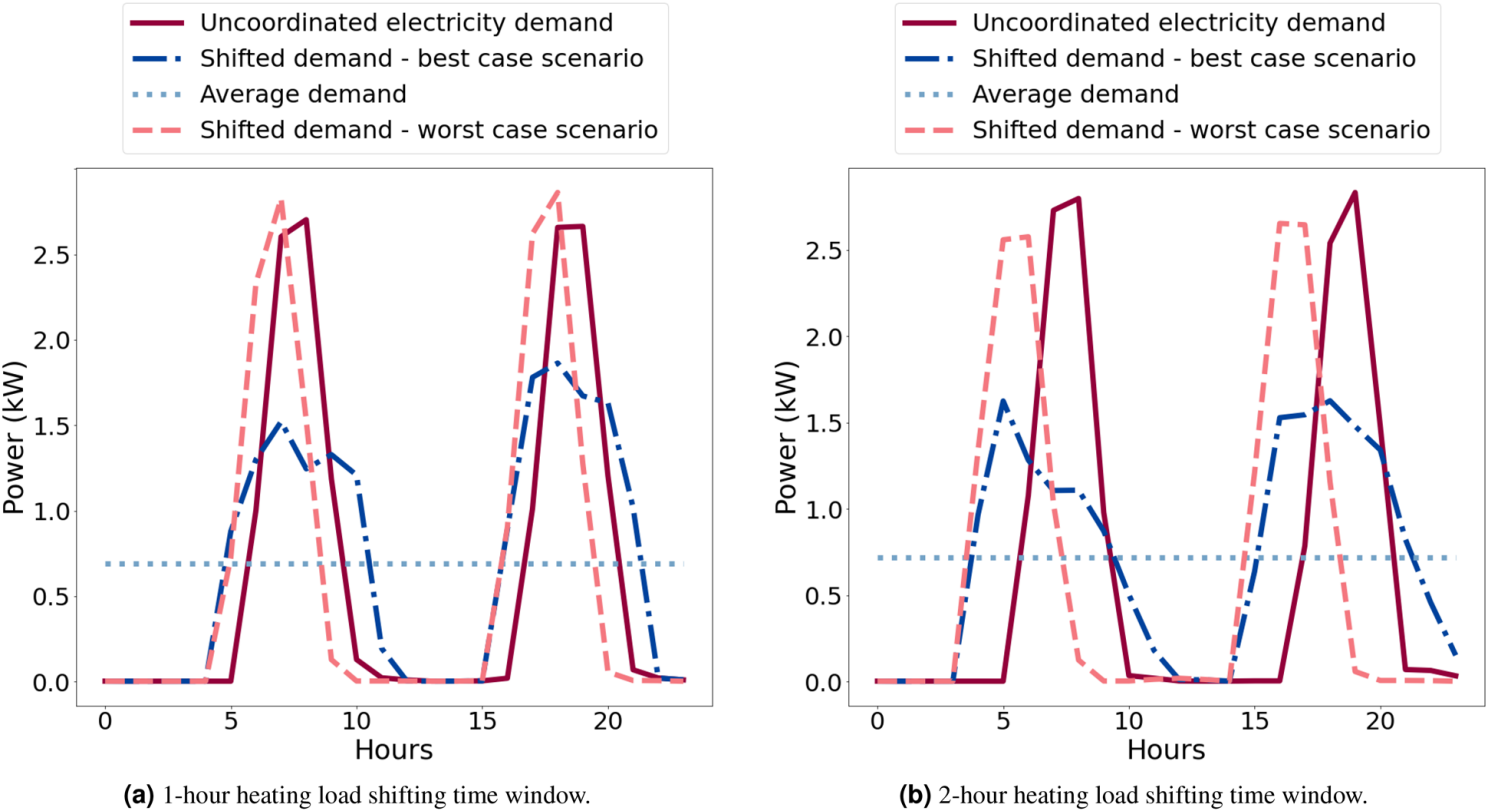
Illustrative examples of 24-h load profiles of a random dwelling for (**a**) 2-h and (**b**) 4-h load shifting time windows. Each graph shows: the peak load coordination schema achieving the most peak demand flattening, the peak load coordination schema achieving the least peak demand flattening, the profiles when no peak coordination schema is applied and constant average demand. Note that the results are based on random simulations without the same seed so they are not directly comparable.

The numerical and experimental tests of such loads using smaller shifting windows of only two or four hours demonstrate the striking possibility that even with the flexibility of just an hour before or after scheduled demand in the timing of these loads, it is possible to obtain around a 40% reduction in peak load demand. This widens to 61% in a ±2-h window; both results being the maximum expected savings. These reductions are associated with a 29% and 31% reduction in ramp rates for the 2-h and 4-h cases, respectively (see illustrative examples in Fig. 12); and an average outage length of 38 and 66 min respectively. The standard deviation for outage lengths for the 4-h case (42 min) is almost two times higher than for the 2-h case (23 min), indicating that households across the sample experience much higher variability of outage lengths as the time window increases. The physical set-up, although still a simple model of the real-world situation, also demonstrated the principle effectiveness of the scheme in a more realistic environment.

Naturally, the drift in indoor temperature caused by a cessation of the heating or cooling system is strongly dependent on the thermal characteristics of the building envelope itself. Highly inefficient envelopes will cause a rapid drift away from comfortable temperatures, resulting in high ramp rates on the network when the system is switched back on. Conversely, well insulated or thermally heavy constructions will result in smaller network ramps (e.g., see Fig. 10). A second factor that can significantly influence this performance is the definition of thermal comfort itself. It is obvious that a narrow definition of comfort, e.g., within a $\pm 2$K tolerance as defined in the international ISO 7730 standard^93^, would result in more rapid excursions of indoor temperatures beyond comfortable levels, during periods of drift. The wider the definition, as for example suggested in recent research^94^ or as adopted in countries such as India^95^, would result in greater flexibility, and hence fewer network peaks. The influence of both these factors merits further investigation.

Additionally, as the grid transforms towards a renewable driven supply mix, likely balanced with storage, there may be a need for demand to track supply rather than to simply flatten peaks. This would be within the capability of the schema presented here by, for example, dynamically changing the target load currently given by the network neighbourhood peak.

In summary, this work has shown that significant reductions in peak loads are achievable through building-to-building load coordination within large networks. The theoretically optimal number of coordinating entities is surprisingly small and analogous to complex natural systems, whilst being network agnostic. These benefits are conferred in the presence of a very small information load at the level of an individual dwelling, i.e., the current load draw in the neighbourhood and the maximum “allowed”. This is in stark contrast to widely adopted DSM schemes that use optimisation techniques and are limited by their dependency on the availability of historical data and forecasts^96,97^. In the future, such coordination will no doubt become essential to manage the huge shifts currently underway in both the supply and demand side of the global energy system, i.e., increasing use of intermittent renewable generation and electricity as the only fuel for cars, space heating and space cooling. The agreement of the numerical simulation and experimental demonstration results is encouraging and strongly suggest that this type of local (or virtual peer-group) load distribution schemes are worth much further investigation.

### Supplementary Information

Supplementary Video S1.

Supplementary Video S2.

## Data Availability

The data and codes that support the findings of this study are available from the following source: https://bitbucket.org/apoghosyan/zedipeaksupression/src/master/.
